# High-quality draft genome sequence of *Sedimenticola selenatireducens* strain AK4OH1^T^, a gammaproteobacterium isolated from estuarine sediment

**DOI:** 10.1186/s40793-016-0191-5

**Published:** 2016-09-08

**Authors:** Tiffany S. Louie, Donato Giovannelli, Nathan Yee, Priya Narasingarao, Valentin Starovoytov, Markus Göker, Hans-Peter Klenk, Elke Lang, Nikos C. Kyrpides, Tanja Woyke, Elisabetta Bini, Max M. Häggblom

**Affiliations:** 1 Department of Biochemistry and Microbiology, School of Environmental and Biological Sciences, Rutgers, The State University of New Jersey, New Brunswick, NJ USA; 2 Institute of Earth, Ocean, and Atmospheric Science, School of Environmental and Biological Sciences, Rutgers, The State University of New Jersey, New Brunswick, NJ USA; 3 Institute of Marine Science, ISMAR, National Research Council of Italy, CNR, Ancona, Italy; 4 Institute for Advanced Studies, Program in Interdisciplinary Studies, Princeton, NJ USA; 5 Department of Environmental Sciences, School of Environmental and Biological Sciences, Rutgers, The State University of New Jersey, New Brunswick, NJ USA; 6 Department of Cell Biology and Neuroscience, Rutgers, The State University of New Jersey, Piscataway, NJ USA; 7 Leibniz Institute DSMZ – German Collection of Microorganisms and Cell Cultures, Braunschweig, Germany; 8 Newcastle University, School of Biology, Newcastle upon Tyne, UK; 9 Department of Energy Joint Genome Institute, Genome Biology Program, Walnut Creek, CA USA; 10 Department of Biological Sciences, Faculty of Science, King Abdulaziz University, Jeddah, Saudi Arabia; 11 Pharmacy Practice and Administration, Ernest Mario School of Pharmacy, Rutgers, The State University of New Jersey, Piscataway, NJ USA; 12 Present address: Department of Biochemistry, Molecular Biology and Biophysics, University of Minnesota, Minneapolis, MN USA

**Keywords:** *Sedimenticola selenatireducens*, *Gammaproteobacteria*, Anaerobe, Selenate respiration, 4-hydroxybenzoate

## Abstract

*Sedimenticola selenatireducens* strain AK4OH1^T^ (= DSM 17993^T^ = ATCC BAA-1233^T^) is a microaerophilic bacterium isolated from sediment from the Arthur Kill intertidal strait between New Jersey and Staten Island, NY. *S. selenatireducens* is Gram-negative and belongs to the *Gammaproteobacteria*. Strain AK4OH1^T^ was the first representative of its genus to be isolated for its unique coupling of the oxidation of aromatic acids to the respiration of selenate. It is a versatile heterotroph and can use a variety of carbon compounds, but can also grow lithoautotrophically under hypoxic and anaerobic conditions. The draft genome comprises 4,588,530 bp and 4276 predicted protein-coding genes including genes for the anaerobic degradation of 4-hydroxybenzoate and benzoate. Here we report the main features of the genome of *S. selenatireducens* strain AK4OH1^T^.

## Introduction

Selenium (Se) is an intriguing element in that microbes actively metabolize it through reduction, oxidation, methylation and demethylation reactions, using some of these to conserve energy. Of particular interest is the process of dissimilatory Se reduction, where the Se oxyanion, selenate [Se(VI)], is sequentially reduced to selenite [Se(IV)] and further to insoluble elemental Se(0). The ability to respire selenate/selenite is comparatively rare, nonetheless, is found in phylogenetically diverse anaerobes [1]. SeRB display a tremendous phylogenetic diversity, and yet the metabolic function seems to be conserved (or alternatively horizontally dispersed) in these unrelated groups. Furthermore, the physiologies of the known selenate-respiring bacteria appear to vary greatly. For example, they are able to couple growth to a wide range of electron acceptors such as arsenate, [2, 3] cobalt oxide (Co(III)) [4], and tellurite [5] to name a few. SeRB have been isolated from a variety of different locations. A few examples are: in California in the San Joaquin Valley [6], from estuarine sediment in NJ [7], from a glass manufacturing plant in Japan [8], and from the dead sea [9].

*Sedimenticola selenatireducens* type strain AK4OH1^T^ (= DSM 17993^T^ = ATCC BA-1233^T^) is a member of the *Gammaproteobacteria* isolated from estuarine sediment for its unique ability to couple the oxidation of aromatic acids to selenate respiration. The genus *Sedimenticola* currently includes seven cultivated strains of which two species have been named and described: *S. selenatireducens* strain AK4OH1^T^, the type strain of the type species for this genus [10], *S. selenatireducens* strain CUZ [11], *S. thiotaurini* strain SIP-G1 [12], *Sedimenticola* sp. strain Ke4OH1 [7], and *Sedimenticola* sp. strain NSS [11]. Here we summarize the physiological features of *Sedimenticola selenatireducens* AK4OH1^T^ and provide a description of its genome.

## Organism information

### Classification and features

**Fig. 1:**
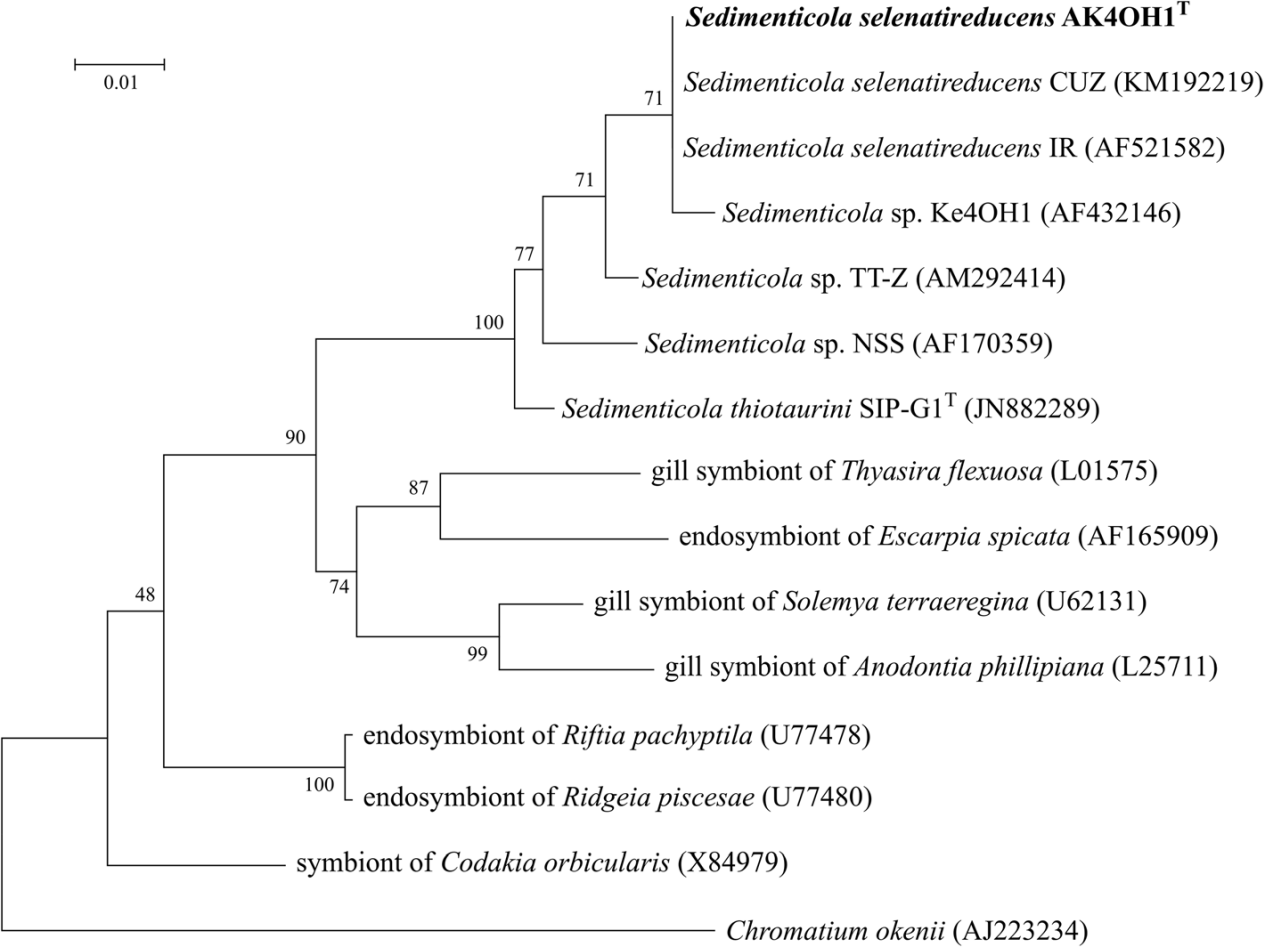
Phylogenetic analysis highlighting the position of *Sedimenticola selenatireducens* strain AK4OH1^T^ relative to its closest neighbors based on the 16S rRNA gene. The evolutionary history was inferred by using the Maximum Likelihood method based on the Tamura-Nei model [29]. The tree with the highest log likelihood (-3985.1130) is shown. The percentage of trees in which the associated taxa clustered together is shown next to the branches. Initial tree(s) for the heuristic search were obtained by applying the Neighbor-Joining method to a matrix of pairwise distances estimated using the Maximum Composite Likelihood (MCL) approach. The tree is drawn to scale, with branch lengths measured in the number of substitutions per site. The analysis involved 15 nucleotide sequences. All positions containing gaps and missing data were eliminated. There were a total of 1276 positions in the final dataset. Evolutionary analyses were conducted in MEGA6 [30]. The strains and their corresponding GenBank accession numbers for 16S rRNA genes are listed in parentheses. The genome accession number and locus tag of strain AK4OH1^T^ are NZ_ATZE00000000.1 and A3GODRAFT_03746. (T = type strain). Bar: 0.01 substitutions per nucleotide position. *C. okenii* was used as an outgroup

*S. selenatireducens* strain AK4OH1^T^ was isolated from estuarine sediment in the New York-New Jersey harbor estuary (40°586′N, 74°207′E) [10]. The position of strain AK4OH1^T^ relative to its phylogenetic neighbors is shown in Fig. 1. *S. selenatireducens* strain CUZ [11] is the closest relative to strain AK4OH1^T^ with a 16S rRNA gene similarity of 100 %, yet interestingly, it has not been found to respire selenate. In addition to these two, there are five other cultivated strains of the genus *Sedimenticola*: *S. thiotaurini* strain SIP-G1^T^ [12], *Sedimenticola* sp. strain NSS [11], and *Sedimenticola* sp. strain Ke4OH1 [7]. The isolate TT-Z (accession number AM292414) [13] groups among the *Sedimenticola* strains (Fig. 1) suggesting that it is part of the *Sedimenticola* genus. The isolate IR (accession number AF521582) groups closely with strain AK4OH1^T^ and strain CUZ, and its position in the phylogenetic tree suggests that it is a member of the *Sedimenticola selenatireducens* species.

**Fig. 2:**
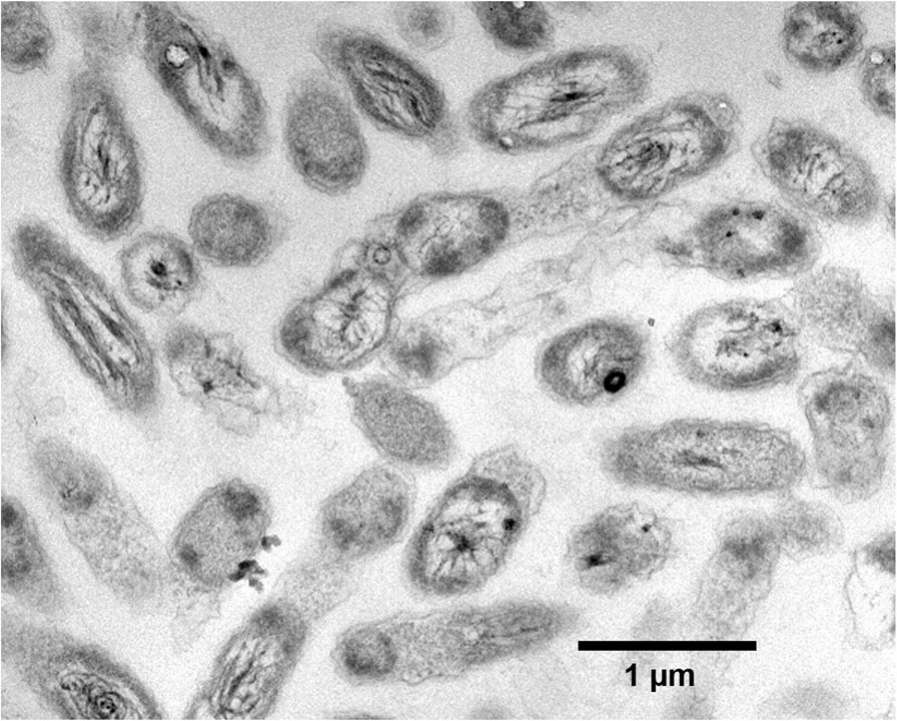
Electron micrograph of cells of *S. selenatireducens* strain AK4OH1^T^. Bar, 1 μm

Cells of strain AK4OH1^T^ are Gram-negative and rod-shaped [10] (Fig. 2 and Table 1). The strain can grow heterotrophically or lithoautotrophically under hypoxic and anaerobic conditions [12]. Motility is observed during early to mid-exponential growth on liquid MB2216 medium, but not in late exponential phase, and cell morphology varies depending on growth conditions [10, 12].

**Table 1:** Classification and general features of *Sedimenticola selenatireducens* strain AK4OH1^T^ according to the MIGS recommendations [18]

MIGS ID	Property	Term	Evidence code^a^
	Classification	Domain *Bacteria*	TAS [31]
		Phylum *Proteobacteria*	TAS [32]
		Class *Gammaproteobacteria*	TAS [33, 34]
		Genus *Sedimenticola*	TAS [10, 35]
		Species *Sedimenticola selenatireducens*	TAS [10, 35]
		Type strain: AK4OH1^T^	
	Gram stain	negative	TAS [10]
	Cell shape	rod (1.5 μm long, 0.5 μm wide)	TAS [10]
	Motility	motile at some growth stages	TAS [12]
	Sporulation	none	TAS [10]
	Temperature range	mesophile	TAS [10]
	Optimum temperature	28 °C	TAS [10]
	pH range; Optimum	7	TAS [10]
	Carbon source	benzoate, 3-hydroxybenzoate, 4-hydroxybenzoate, acetate, formate, pyruvate, methyl-pyruvate, L-lactate, D- and L-malate, propionate, fumarate, succinate, methyl-succinate, bromo-succinate, p-hydroxyphenylacetic acid, cysteine	TAS [10, 12]
MIGS-6	Habitat	estuarine sediment	TAS [10]
MIGS-6.3	Salinity	1.1-2.3 % NaCl (w/v)	TAS [10]
MIGS-22	Oxygen requirement	anaerobe-microaerophile	TAS [10, 12]
MIGS-15	Biotic relationship	free-living	TAS [10]
MIGS-14	Pathogenicity	unknown	NAS
MIGS-4	Geographic location	Hudson River estuary, Arthur Kill, intertidal strait NY/NJ, USA	TAS [10]
MIGS-5	Sample collection	1995	TAS [10]
MIGS-4.1	Latitude	40°586′N	TAS [10]
MIGS-4.2	Longitude	74°207′E	TAS [10]
MIGS-4.3	Depth	surface sediment	TAS [10]
MIGS-4.4	Altitude	sea level	TAS [10]

^a^ Evidence codes - IDA: Inferred from Direct Assay; TAS: Traceable Author Statement (i.e., a direct report exists in the literature); NAS: Non-traceable Author Statement (i.e., not directly observed for the living, isolated sample, but based on a generally accepted property for the species, or anecdotal evidence). These evidence codes are from the Gene Ontology project [36]

Strain AK4OH1^T^ is able to utilize benzoate, 3-hydroxybenzoate, 4-hydroxybenzoate, acetate, formate, fumarate, L-lactate, D- and L-malate, pyruvate, methyl-pyruvate, propionate, succinate, methyl-succinate, bromo-succinate, p-hydroxyphenylacetic acid, α-ketoglutaric acid, arabinose, lyxose, ribose, xylose, D-galactonic acid-γ-lactone, α-hydroxy-glutaric acid-γ-lactone, L-alanine, L-glutamic acid, L-serine, tyramine, and phenylethylamine [10, 12].

#### Chemotaxonomic data

The predominant cellular fatty acids in strain AK4OH1^T^ are C_16:0_ (61.9 %), C_16:1_ ω7c (14.4 %), C_18:0_ (8.4 %), and C_18:1_ ω7c (7.2 %) [10].

## Genome sequencing information

### Genome project history

*S. selenatireducens* strain AK4OH1^T^ was selected for sequencing in 2011 based on its phylogenetic position [14, 15] and is part of the study Genomic Encyclopedia of Type Strains, Phase I: the one thousand microbial genomes project (KMG-I) [16]. The goal of the KMG-I study was to increase the coverage of sequenced reference microbial genomes [17]. The Quality Draft (QD) assembly and annotation were made available for public access on June 18, 2014. Table 2 presents the project information and its association with MIGS version 2.0 compliance [18]. The NCBI accession number for the Bioproject is PRJNA165429. The genome accession number is ATZE00000000.1 consisting of 41 contigs (ATZE01000001-ATZE01000041) and 37 scaffolds.

**Table 2:** Project information

MIGS ID	Property	Term
MIGS 31	Finishing quality	Level 2: High-Quality Draft
MIGS-28	Libraries used	Illumina std PE IIOC
MIGS 29	Sequencing platforms	Illumina
MIGS 31.2	Fold coverage	273×
MIGS 30	Assemblers	ALLPATHS v. R37654
MIGS 32	Gene calling method	Prodigal 2.5
	Locus Tag	A3GO
	Genbank ID	ATZE00000000.1
	GenBank Date of Release	06/18/14
	GOLD ID	Gp0013295
	BIOPROJECT ID	PRJNA165429
MIGS 13	Source Material Identifier	AK4OH1^T^
	Project relevance	Bioremediation, environmental, biogeochemical cycling of Se, Genomic Encyclopedia of Bacteria and Archaea (GEBA)

### Growth conditions and genomic DNA preparation

*S. selenatireducens* strain AK4OH1^T^ was grown in mineral salt medium at 28 °C with 10 mM Na_2_SeO_4_ as electron acceptor and 250 μM 4-hydroxybenzoate as carbon source, as previously described [10]. Genomic DNA was isolated from 0.5 g of cell paste using JetFlex Genomic DNA Purification Kit (GENOMED) as recommended by the manufacturer.

### Genome sequencing and assembly

Sequencing was achieved using an Illumina [19] platform using a std paired-end library obtaining 273× fold coverage. The sequencing was done at the DOE Joint Genome Institute. ALLPATHS assembly software [20] was used to obtain 41 final contigs. Quality check and assembly statistics were performed at JGI. The raw sequences were screened against contaminants and 0.1 % of the reads were removed.

### Genome annotation

Gene calling was performed using Prodigal 2.5 [21]. The genome sequence was analyzed using the Joint Genome Institute IMG system [22]. Ribosomal RNAs were predicted based upon sequence similarity, using BLAST, against the non-redundant nucleotide database and/or using Infernal and Rfam models. tRNA genes were found using tRNAscan-SE [23]. The predicted CDS were searched using the NCBI non-redundant protein database. The major metabolic pathways and predicted protein set were searched using KEGG, SwissProt, COG, Pfam, and InterPro protein databases implemented in the IMG. Additional gene prediction analysis and manual functional annotation were performed within IMG and using Artemis software (release 13.0, Sanger Institute).

## Genome properties

The high quality draft genome sequence consists of 37 scaffolds that account for a total of 4,588,530 bp with a 56.6 % G + C content. In total, 4331 genes were predicted, 4276 of which are protein-coding genes, 55 RNA genes, and no pseudogenes. The majority of the predicted genes (79 %) were assigned a predicted function. The properties and statistics of the genome are summarized in Table 3 and Table 4.

**Table 3:** Genome statistics

Attribute	Value	% of Total^a^
Genome size (bp)	4,588,530	100.00
DNA coding (bp)	4,041,165	88.07
DNA G + C (bp)	2,597,447	56.61
DNA scaffolds	37	100.00
Total genes^b^	4331	100.00
Protein coding genes	4276	98.73
RNA genes	55	1.27
Genes with function prediction	3440	79.43
Genes assigned to COGs	2832	65.39
Genes with Pfam domains	3595	83.01
Genes with signal peptides	383	8.84
Genes with transmembrane helices	1143	26.39
CRISPR repeats	1	-

^a^ The total is based on either the size of the genome in base pairs or the total number of protein coding genes in the annotated genome

^b^ no pseudogenes found

**Table 4:** Number of genes associated with general COG functional categories

Code	Value	%age	Description
J	205	6.48	Translation, ribosomal structure and biogenesis
A	1	0.03	RNA processing and modification
K	180	5.69	Transcription
L	117	3.70	Replication, recombination and repair
B	2	0.06	Chromatin structure and dynamics
D	41	1.30	Cell cycle control, Cell division, chromosome partitioning
V	66	2.09	Defense mechanisms
T	244	7.71	Signal transduction mechanisms
M	160	5.06	Cell wall/membrane biogenesis
N	120	3.79	Cell motility
U	49	1.55	Intracellular trafficking and secretion
O	207	6.54	Posttranslational modification, protein turnover, chaperones
C	339	10.71	Energy production and conversion
G	116	3.67	Carbohydrate transport and metabolism
E	244	7.71	Amino acid transport and metabolism
F	57	1.80	Nucleotide transport and metabolism
H	166	5.24	Coenzyme transport and metabolism
I	148	4.68	Lipid transport and metabolism
P	187	5.91	Inorganic ion transport and metabolism
Q	76	2.40	Secondary metabolites biosynthesis, transport and catabolism
R	211	6.67	General function prediction only
S	175	5.53	Function unknown
-	1499	34.61	Not in COGs

The total is based on the total number of protein coding genes in the genome

## Insights from the genome sequence

The respiratory flexibility of anaerobic prokaryotes allowing them to employ different terminal electron acceptors for respiration enables these organisms to thrive in dynamic redox environments. Among the enzymes that catalyze oxidation-reduction reactions of metals and metalloids are those that are highly conserved and belong to the DMSO reductase family [24]. Key members of the DMSO family of reductases, which transfer electrons to a variety of substrates that act as terminal electron acceptors for energy generation, are nitrate reductases (Nar, Nap, Nas), arsenate reductase (Arr), selenate reductase (Ser), and chlorate reductase (Clr), among others.

*S. selenatireducens* strain AK4OH1^T^ can use nitrate, nitrite and selenate as the terminal electron acceptors for anaerobic growth, while using the electron donors acetate, lactate, pyruvate, benzoate, 3-hydroxybenzoate, and 4-hydroxybenzoate [10]. Chlorate and perchlorate can be used as electron acceptors when peptone is used as an energy source [12]. (Micro-)aerobic growth with oxygen as electron-acceptor and peptones as electron-donor is also detected [12]

**Fig. 3:**
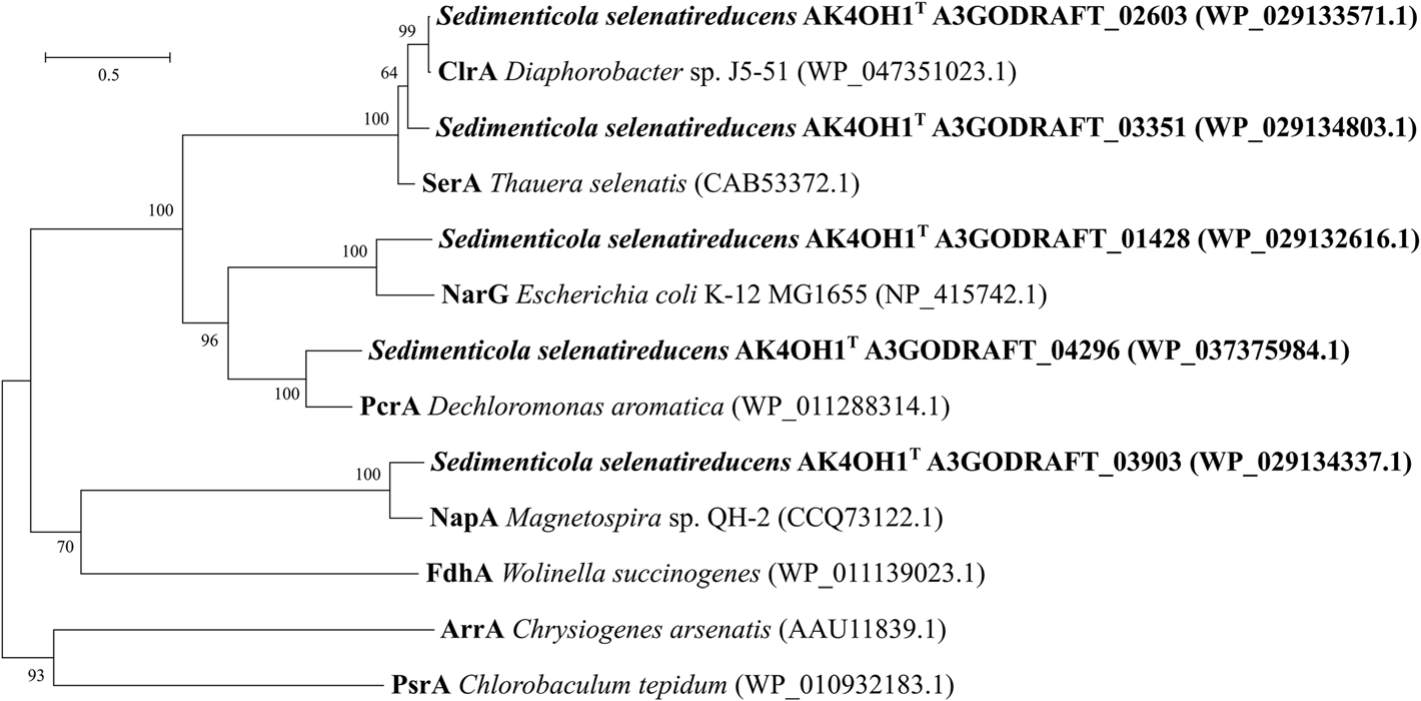
Phylogenetic analysis highlighting the relation of *Sedimenticola selenatireducens* strain AK4OH1^T^ genes to known DMSO reductases by Maximum Likelihood method. The evolutionary history was inferred by using the Maximum Likelihood method based on the JTT matrix-based model [37]. The tree with the highest log likelihood (-17325.9218) is shown. The percentage of trees in which the associated taxa clustered together is shown next to the branches. Initial tree(s) for the heuristic search were obtained by applying the Neighbor-Joining method to a matrix of pairwise distances estimated using a JTT model. The tree is drawn to scale, with branch lengths measured in the number of substitutions per site. The analysis involved 13 amino acid sequences. All positions containing gaps and missing data were eliminated. There were a total of 724 positions in the final dataset. Evolutionary analyses were conducted in MEGA6 [30]. GenBank accession numbers are listed in parentheses. Bar = 0.5 substitutions per nucleotide position

Within the AK4OH1^T^ genome, there are several likely DMSO reductases. Figure 3 shows the grouping of AK4OH1^T^ genes with closely matching, known, DMSO reductases. A3GODRAFT_03903 groups closely with the NapA, from *Magnetospira* sp. QH-2. A3GODRAFT_01428 clusters together with the NarG of *Escherichia coli* K-12 MG1655. Both of these genes are organized in gene clusters similar to known *nap* and *nar* operons [25]. BLAST searches of the AK4OH1^T^ genome using arsenate reductases showed no genes with significant similarity. This agrees with strain AK4OH1’s inability to respire arsenate [10]. A3GODRAFT_02603 and A3GODRAFT_03351 from strain AK4OH1^T^ cluster closely with the chlorate reductase from *Diaphorobacter* sp. J5-51 and with the selenate reductase from *Thauera selenatis*. A3GODRAFT_02603, which groups closest with ClrA, resembles the gene organization of a *clr* operon [26]. While the only well-studied respiratory selenate reductase, *serA,* is from *Thauera selenatis*, A3GODRAFT_03351 and its neighboring genes follow the same organization as found with *serABDC* [27]. Gene A3GODRAFT_04296 clusters together with the perchlorate reductase from *Dechloromonas* *aromatica,* and appears to have the same gene organization as a *pcr* operon [28].

## Conclusions

The complete genome of the estuarine bacterium *Sedimenticola selenatireducens* AK4OH1^T^ provides a stronger foundation from which to learn more about the process of dissimilatory selenate reduction. As AK4OH1^T^ was the first organism isolated capable of coupling the respiration of selenate to the oxidation of benzoic acids, its genome also provides a starting point for learning more about this unique capability.

## Abbreviations

DMSO: Dimethyl sulfoxide
SeRB: Selenate reducing bacteria
